# Book Reviews

**Published:** 1818-12

**Authors:** 


### CRITICAL ANALYSIS

### *OF RECENT PUBLICATIONS,*

IN THE

### DIFFERENT BRANCHES OF MEDICINE, SURGERY, &c.

*Remarks on the Pathology and Treatment of Typhous Fever; by* Albert Henry Callanan, M.D.—8vo. Cork, 1818.

IT is with pleasure we notice every attempt to throw additional light on a subject of so much contention amongst the medical profession as what is usually denominated Typhous Fever, in hopes that, by contrasting the conflicting opinions, and drawing a fair comparison between them, much may be done towards accomplishing a settled and general opinion, as relates to the nature and treatment of the disease before us, as well as fevers of all denominations.

Dr. Callanan observes, "I am influenced by no other hope than that, in passing the ordeal of scrutiny, a candid consideration may be given to them. Fortunately they are not peculiarly mine. The subject has of late been taken up with no common degree of ability by several British physicians, and treated with all the force of talent and ingenuousness;" and he assigns to Dr. Armstrong the first place among those who have had similar views to his own on the subject, observing they were acted upon in his own practice for some years back.

In the work before us, the author has endeavoured to concentrate his ideas, and gives us a hope that he will resume the subject at some future period.

After paying a proper compliment to those who have contributed to the progressive improvements of time, Dr. Callanan attributes much to our military and naval establishments, in tropical climates, reviving the practice of Sydenham, which had been unfortunately brought into disrepute; and, as it were, enters upon the ideas he entertains respecting typhous fever. "The fevers of those regions (tropical climates) are ushered in with all the symptoms of vascular excitement, attended with the consequent effect of unequal distribution of the blood, as indicated on dissection, by inflammatory and congestive appearances in the various organs, but more particularly in the brain and hepatic system."

Fully agreeing with Dr. Armstrong's classification and arrangement, he has adopted his distinguishing terms—*simple*, *inflammatory*, and *congestive typhus.*

We would ask, does an identical and an individual disease admit of any other distinctions than such as relate either to its intensity or to its progressive stages? It is to be apprehended, that the multiplication of distinctions of individual diseases, of any class or genus whatever, has led to much confusion, and consequent error, in medical practice; and perhaps it may be added, that this proposition is most pointedly exemplified in those diseases denominated fevers. Dr. Callanan states his reasons, however, for so fully coinciding with Dr. Armstrong—

"Because I think it unfolds the most correct, and (to use a fashionable term) the most *legitimate* view of the phenomena of fever, with which I am acquainted. The plan of treatment, too, as by him laid down, has this advantage—that it confines its operations to inductions drawn from plain and ostensible premises, and leads the physician to adopt indications of cure founded on specific and definite views, rather than on the vague and general plan hitherto pursued by medical men, of taking the characters and treatment of disease from the indefinite and unmeaning *verbiage* of some of the earlier medical writers, sanctioned by later names of (it must be admitted) established reputation. A blind and unthinking adherence to what our instructors have taught us is often a principal source of imperfection in many sciences, but more particularly in that of medicine. What, for example, can be more absurd than to hear many practitioners of the present day asserting that unity of character should be attached to typhous fever,—or, in other words, that it is always a disease of debility and putrescency, requiring no modification of treatment, save the quantity or quality of stimulus which the urgency of symptoms may seem to call for. This, however, and other similar dogmas of the more imperfect periods of our art, are falling fast into merited disrepute and oblivion; and are giving place to more solid and philosophical trains of thinking and reasoning than the substitution of terms for explanations."

A proper selection of appropriate terms is, at the present time, essential to the establishment of a solid system of medical science at large, and more especially in the department of nosology. If technical terms do not raise the same image in the minds of those who hear as exists in the minds of those who teach, the language must be defective; and, howsoever such terms may be contorted, and artfully connected by intermediate phraseology, they will never be capable of conveying correct and just definitions and explanations, sufficiently uniform in their general aspect to be viewed exactly alike by any two individuals. Terms, in medical language, constitute the nomenclature of medical science, and they require to be of chaste derivation and of determinate import; each term will then, when fully understood in its import and application, amount to an explanation, or to description; for description consists of an explanation of technical terms, constituting nomenclature.

As before stated, typhous fever is divided by Dr. Callanan, in concurrence with Dr. Armstrong's division of it, into *simple*, *inflammatory*, *congestive*, and *subacute;* and he gives the symptoms of the simple typhous fever in the following order:—

"The accession of typhus in its more simple form is ushered in with the following symptoms:—The patient complains of langour and lassitude, slight chills, or distinct rigors; followed by a sensation of uncomfortable heat,—pain or confusion of the head,—nausea, or vomiting,—an irregular state of the bowels, more frequently inclining to costiveness than to the opposite extreme,—loss of appetite,—unquiet sleep,—a torpid state of the mental functions. Sometimes, however, there is great apprehension of death, and the patient sighs frequently. For the most part, the skin is dry, but sometimes it is moist. The tongue is white, but its edges are generally red; sometimes too it is brown or black, even from a very early period of the disease. The eyes have a dull watery appearance, with small red vessels running on the adnatæ, and sometimes we observe a black colour of the cellular substances under them. This appearance is oftener remarked in females than in men. In the earlier stages, the urine does not often vary from the natural colour: in some instances, however, it will be found turbid or pale; in the latter case, it is often passed in very large quantities, and is said to be an unfavourable symptom. During this period of the complaint, the pulse ranges from 86 to

108. Its volume is sometimes increased, and sometimes diminished: under the latter appearance, it will often be found to possess an asperity and labour in its contractions, which give to the practitioner the idea of something clogging the motion of the vessel, from which it in vain endeavours to free itself. After the continuation of these appearances for an uncertain period of from one to four days, the complaint begins to develop itself more clearly, and to assume a more active train of operation. The countenance rather brightens, and becomes florid; restlessness and parched skin are now observed; the foulness of the tongue increases; the pulse becomes more expanded in its volume, and increases in frequency; and all the symptoms of re-action come on in quick succession. Nature is now struggling fast. The venous and arterial systems are endeavouring to accommodate themselves to this new order of things, and the circulation of the blood is the channel through which the patient's destiny is to come. If, from constitutional energy, or other causes with which we are unacquainted, the balance of distribution in that quarter be equipoised, though the complaint may perhaps continue to a protracted and uncertain period, its aspect will be mild, and its character uncomplicated. When, however, the reverse happens, and that error or unequal* distribution be established in the circulating system, new and more formidable struggles will take place; and whether temporary or permanent mischief shall follow will depend upon the intensity and duration of the excitement, and the impressions made upon the organs affected. Should the vital organs be the points to which this irregularity is directed, their functions will be clogged or perverted in their operations, and the physician will have to contend with all the formidable and overwhelming symptoms necessarily following a state of such general and local tumult."

It is of the utmost importance in nosology, however much it may have been disregarded, to be exactly chronological in the enumeration of symptoms; they should succeed each other in the order of time in which they are discovered, not take precedency according to their more obvious or afflicting characters. Languor and lassitude are symptomatic of the existence of a diseased state, and consequently of derangement of function of either the membranous or muscular solids externally situated,—and this state is inflammatory; it is the idiopathic disease, *membranous* or *muscular inflammation*,—we will suppose *membranous*; and the slight chills or distinct rigors, followed by a sensation of uncomfortable

* Would it not appear, from the following quotation, that Baglivi saw the full force of this state of things, when he says:—Postremo febrium curationem certius non absolvi, quam in *continendo sanguinem intra eos limites, ut neque hinc plus æquo gliscat: neque illinc nimium torpeat?*—Praxeos Medicæ, liber ii.

heat, are the consectaria consequences, symptomatic of local membranous inflammation. If the attack be slight, the disease may, after a short period, altogether retire; otherwise, it will assume a remittent type, and the inflammation will not be limited to its primary situation, but will extend throughout the whole of the same order of solid, affecting some parts more than others,—as the pleura, the peritoneum, &c. —or, if the attack be excessively severe, the disease will keep increasing in all its forms and consequences; that is, the local inflammation will make rapid progress, and the constitutional sympathies will be commensurate: in other words, the fever will correspond with the local inflammation in intensity and in progression. It will be easily conceived that when the original attack is upon an internal membrane, that the symptoms, both as they relate to the local affection and to the constitutional sufferings, will be more afflicting according to the situation of the local inflammation,—such as pain or confusion of the head, nausea or vomiting, an irregular state of the bowels, &c.; but still the type of the disease will be dependent upon the degree of its intensity, let the primary attack of inflammation be where it may; and, moreover, a slight attack, with considerable remissions, may be, and very often is, by improper treatment, so much aggravated as to assume a progressive type; that is, the local inflammation extending, and proceeding to the destruction of the parts which are the subjects of it, by their own excess of action, without any evident remissions of pain or fever.

The order, then, in which the symptoms of typhous fever should be laid down is—a reference to some membranous part in pain, evidently inflamed, by the presence of those symptoms by which local inflammation is distinguished,—namely, slight chills or distinct rigors, followed by a sensation of uncomfortable heat, &c. with impaired or disturbed function of the part particularly aggrieved.

These are the first signs of what is denominated typhous fever,—more properly, remitting membranous inflammation; and it is pity it has not obtained a denomination expressive of its real nature and character. Such an appellation would do away with hypothetical opinions; would render that multiplication of accidental symptoms in the description of it unnecessary; would banish the alarms inseparable from the commonly-received notions of debility and putrescency; and have a tendency to establish a settled general method of treatment, founded upon solid and just principles.

Dr. Callanan proceeds—

"In so complicated and nicely-balanced a machine as the human frame, the power of resisting morbid impressions will probably depend much upon *Idiosyncrasy*. Exemption from and aptitude to disease, are circumstances in pathology so little understood, as to cause great imperfection in that part of medicine which constitutes one of its first *desiderata*,—namely, the prevention of disease. But to return,—the transition from the simple to the more complicated forms of typhous fever is often as sudden as it is unexpected on the part of the physician.

"Several instances of this alarming accession have occurred to me in the course of my practice, but more particularly during the prevalence of the present epidemic."

—and gives a case illustrative of the simple typhus making a sudden transition into one of the more complex forms:

"A young man, after exposure to cold, was attacked with all the symptoms of mild typhus. I saw him, for the first time, on the eighth day of his illness: his pulse was 88, and soft,—skin cool, and moist; he was covered with small florid petechiæ,—his bowels were open,—he was free from delirium; in short, all the functions appeared to be natural and regular. I directed infusion of roses, with sulphuric acid, to be taken at intervals; and the patient to be supported with gruel, arrow-root, and roasted apples. The young man's father said, he would come for me again if the patient should get worse. On the following day, a female relative of his, who acts as nurse-tender in this city, came to see him: on observing the petechiæ, she alarmed his parents, and said that *wine* was the only cure for a *spotted fever*, and that without it the patient could not recover. Accordingly, he was plied with mulled wine, alternated with draughts of boiled porter and whiskey. On the second day after, I was requested to see him again, and found him labouring under all the symptoms of highly inflammatory typhus. The eye was wild; pulse 136, wiry and full; the delirium almost amounted to frenzy; great oppression about the precordia; bowels costive; subsultus tendinum approximating to general convulsions; and, what will appear strange with these symptoms, the patient was absolutely *deluged in perspiration*. Calculating upon this last appearance, I thought some good may arise from it, and determined to follow up the indication Nature appeared to be pointing at; I therefore directed twelve grains of calomel, with two of antimonial powder, to be given immediately, and that tepid barley-water may be taken as drink. I fixed upon a period of two hours for my return. I had scarcely left my patient an hour, however, when a messenger came to request my immediate presence,—as torrents of blood, he said, were flowing from his nose. By the time I could get to him (about a quarter of an hour), the blood had ceased to flow, but not before he lost full thirty ounces. The skin was now dry. The carotid arteries were beating with violence; the countenance less florid, but the eyes were suffused and wild; the pulse was 128, and hard; petechiæ numerous, and subsultus still considerable; respiration laborious; bowels still confined. A purgative enema was ordered; and, while I was impressing upon his mother the necessity of removing him to a more airy apartment than that in which he was, the patient, in a convulsive effort, sat erect in his bed, with a countenance expressive of great anxiety. The bleeding from the nose now returned; and, as the pulse still continued uninfluenced by the loss he had already sustained, I determined on venesection,—and accordingly fifteen ounces of blood were in a short time taken from his arm. The enema before ordered was now given; and the calomel bolus was directed to be repeated at eight o'clock in the evening, being a period of five hours from his taking the first.

"The bleeding had an almost instantaneous effect upon the hæmorrhage, and indeed upon the whole system. The pulse fell to 100 within half an hour; and shortly after the patient showed some tendency to composure, if not to sleep,—from which, however, he was soon disturbed, by the operation of the enema, which produced two evacuations. As matters were in this state, and as twenty-four grains of calomel would have been taken, and forty-five ounces of blood lost, I determined to await the issue of the night without further interference.

"On visiting my patient the following morning, I found him asleep; pulse 90 and soft, respiration and heat almost natural; petechiæ still distinct, but less numerous. On awakening him, he answered my questions with perspicuity and point. During the night he had passed twelve or fourteen dark-coloured fœtid stools, which relieved the abdominal soreness considerably, and entirely removed the fullness there. He was now very languid, and light beef-tea was ordered for common drink, which he relished. He passed the day quietly; but towards night his pulse rose to 100, and the soreness and tension of the abdomen returned. Six grains of calomel were now ordered; and, in an hour after, three ounces of infusion of tamarinds and senna were given. In about two hours after, his bowels were affected five or six times; comfort and quiet returned, and he slept during the remainder of the night.

"On the following day, his pulse was 88 and soft, his respiration natural, petechiæ nearly gone, and he had a general moisture on his skin. He was slightly salivated. From this period his convalescence was established, and his recovery rapid."

This is an instance, and a striking one, of the true inflammatory typhus supervening to the mild or simple typhus,—owing to the improper use of stimulants; and Dr. C. further adds, that transitions of this nature will frequently occur without any obvious cause. But all this only tends to prove that every distinction of typhus is identically inflammation of some definable solid, and that state is denoted by certain evidences called symptoms, particularly those affecting the system at large, called fever; and that the transition from simple to inflammatory typhus is merely an aggravation of local inflammation, and consequently an extension of it:

or, in other words, it is a low degree of intensity, known by distinct remission of both the local affection and the corresponding remissions of the general sufferings being heightened into a greater degree of intensity, with indistinct remissions in rapid succession, approaching to a progressive type. And, as Dr. Callanan observes, such transitions may take place at any period of the disease,—that is, any degree of inflammation may, by improper treatment, be raised to the progressive type ; and it will be here properly observed, that what is usually called typhus gravior is merely symptomatic of the progressive type of inflammation of some distinct animal solid—of any solid whatever, excepting glands and adipose substance.

Dr. Callanan gives an instance of the sudden supervention of inflammatory symptoms to the mild form of the complaint, at an advanced period of it.

"My respected friend, Dr. John Milner Barry, was in attendance with me;—the subject of it was a gentleman of a full sanguine temperament. The origin and progress of this case were marked by the usual symptoms of mild typhus. Some determination of blood to the head, indeed, showed itself early in the disease, but was to all appearance effectually removed by the application of twenty leeches to his forehead and temples. Petechiæ, slight subsultus, and the ordinary train of what are denominated symptoms of putrescency, set in about the ninth day. The tongue became foul, and the abdomen tense and hard; but the pulse remained full, and at 116. Purgatives were used rather freely, and stimuli were withheld. From this plan of treatment great mitigation of these appearances was obtained, and the disease seemed to re-assume a mild character. About the thirteenth day, notwithstanding, violent delirium, general subsultus, and grinding of the teeth, suddenly came on. The pulse rose to 136, and the skin became hot and parched: ten leeches were applied to the temples, and about eight ounces of blood were taken in a very short time from the back of the neck by scarifications and cupping-glasses. Calomel and infusion of senna were then given in liberal doses, and all the untoward symptoms yielded in a few days. Musk and hyosciamus were afterwards had recourse to, with the view of allaying much nervous irritability, which remained for some time after, and appeared to answer this indication very well. During the progress of this case, though a protracted one, the use of wine was totally contra-indicated. Small portions were once or twice tried under circumstances apparently favourable to its use, but they very shortly produced great and alarming excitement."

This case shows that the medical treatment, in all the stages of fever, should be regulated by existing symptoms, and that we should not be awed by fears of debility and putrescency. The practice in this instance was truly judicious, and ought to stand as an example. Probably, had the opposite method of treatment been employed, the case would have terminated fatally.

"There can be no two positions in pathology better established, nor more at variance with each other, than the effects which stimuli and blood-letting, with its concomitant evacuants—such as purging, low diet, &c. produce upon the animal frame. Is it then rational to suppose, that the same object can be attained through the medium of agents so totally dissimilar and discordant in their effects. I am prepared to hear it said, that similar appearances have been successfully treated by a practice the reverse of that inculcated in the history of these two cases. This is the point at issue,—the pivot upon which every thing turns; and let it be candidly considered."

It is by proper attention to the types of inflammatory affections, that we obtain a just diagnosis, prognosis, and the indications of treatment; as well as explain why apparently-similar symptoms will frequently be met with in the progress of typhous fever, and that they would be exasperated by evacuants, and alleviated or removed by wine, camphor, and opium,—and that even before the excitement of simple typhus has produced a state of collapse; for daily experience shows that the disease under consideration is viewed only in three forms or types—intermittent, remittent, and progressive. The identity of the disease is the same—inflammation, but with different degrees of intensity.

"In some constitutions, the same exciting causes of disease will give rise to different modifications of the same complaint, and evince their versatility of power by producing, in different individuals exposed to their influence, the mildest and the most aggravated effects. A striking instance of this came within my observation very recently.—The mother of a large family was attacked with mild typhus; two of her daughters and one of her sons, who were in constant attendance upon her, caught the complaint. In one of the daughters it proved mild; in the son very severe; and to the second daughter it proved fatal on the sixth day. In this case, convulsions and apoplectic stertor set in from the second day, and continued to the morning of the sixth, when death closed the scene, under all the appearances of compressed brain."

This Dr. Callanan considers an instance of pure congestive typhus.

When the local inflammation is in the meninges of the brain, it sometimes resolves by effusion, constituting hydrocephalus. But, when the substance of the brain itself, or glandular substances within the head, are the subjects of the disease, abscess is to be apprehended: in either case, there will be pressure on the brain, and consequently apoplectic stertor. The former of these states is fully illustrated in the case adduced by Dr. Callanan.

"An example of this termination, in a case of inflammatory typhus, I had an opportunity of ascertaining by dissection —In the year 1811, a coasting trader from Wexford put into the harbour of Ring, near Clonakilty, where I then resided. The master came on shore, and drank freely for a few days. He was then seized with all the symptoms of inflammatory typhus, under great determination to the head. Petechiæ and subsultus to an enormous degree soon made their appearance; and the complaint continued to the sixteenth day, when he died. Frequent and general convulsions preceded this event for two days. The head was opened by Mr. Folliot, of Clonakilty. On removing the cranium in the usual way, a fluctuation was observed under the dura mater, and, on puncturing this membrane, about four ounces of clear serum rushed out. The vessels on the surface, and in the substance of the brain, were turgid with dark-coloured blood. On descending to the ventricles, about six ounces more of serum were collected from these cavities. In this case, the effects of inflammation were more than usually evident, even in instances of acute hydrocephalus."

Dr. Callanan gives a fair description of the symptoms of inflammatory typhus, acknowledging that "they are the same as the mild species, but in a severer degree." He gives his reasons for considering the jail-fever, described by Dr. Huxham and others, an example of pure congestive typhus.

"It must be admitted that virulent contagion will very frequently produce the sudden and overwhelming effects which this form of typhus assumes, and that it gives rise to these effects by its peculiar power of oppressing or destroying the energy of the nervous system, and thus weakening the powers and functions of the heart and arteries, is, I think, equally rational as an assumption. To some, the use of the term assumption here may appear a bad foundation, from which to deduce practical conclusions in medicine. To me, however, it seems sufficiently strong; for, in the present state of our knowledge on many of the laws of vitality, but more particularly on the subject of nervous pathology, we cannot assign to several of the sources of our inductions more than an existence gratuitously admitted; and, though this mode of reasoning may not be founded on the basis of logical regulation, as established by the schools, Nature, in many of her operations, requires not this formality from her followers. It is true she will sometimes hide from them her principles of action; but, nevertheless, she will make it self-evident and unquestionable, that she acts not from an unmeaning impulse; and it will be found that many inferences thus drawn will stand the test of the most rigid scrutiny, because they are found universally prevalent in some of the most familiar, but most inexplicable, operations of animal and vegetable life."

Is not the first obvious effect of virulent matter, applied to the living animal fibre, always inflammation, whether it be variolous, rubeolous, syphilitic, or even simply putrid? and is not the progress of them all, both as regards the local affection and the constitutional sympathies, altogether of that character?

It admits of a doubt whether there be any such power in nature as a sedative, excepting what has the property of abstracting some one of the elements, or one of the principles of animal life. Thus, for example, caloric is one of the essential attributes of animal life; therefore, if caloric be abstracted from the living fibre beyond certain limits, that fibre will have its functions diminished, or even destroyed, according to the degree of abstraction of caloric. Again,—vascular action is essential as a constituent part of animal life; and it may be diminished, or even abolished, by pressure. But it appears contradictory to attribute a directly-sedative property to virulent contagious matter: it may be viewed as an indirect sedative, producing oppression or destruction of the nervous energy, from having first excited it to a degree above the limits of health,—on the same principle as a sudden vivid flash of light produces paralysis of the retina.

"It appears to me, however, that this variety of typhus is very frequently the concomitant of the inflammatory species; either as assuming its most violent form at its first onset, or as having its character aggravated in the course of its progress by injudicious treatment."

The two distinctions are not necessary; indeed, they are the same identical affection—inflammation; and exhibit no other varieties than what may be attributed to intensity and duration.

We transcribe a note, which fully accords with our own views of the subject, and leads to important practical deductions.

"Upon the dissection of those who have died of fevers, there have been found inflammation, abscess, and mortification, in the brain and other viscera. These symptoms, however, are only to be considered as the consequences, and not the cause of the disease. Such appearances are very generally found upon examining the bodies of those who die of putrid fevers. We are informed by Sir John Pringle, that, when the jail-fever proved fatal, it terminated in actual mortification of some part; that the intestines, in particular, were apt to mortify; and that abscesses were found in the brain. In the putrid remitting fever of Minorca, Dr. Cleghorn found the intestines of those that died either entirely or partly mortified, and partly inflamed. Bartholine observed the same appearances in those who died of the epidemic which raged at Copenhagen in 1652. From numerous dissections of those who died of the plague at Marseilles, and of the malignant fever at Rouen, some of the viscera were always found in a gangrenous or inflamed state. See a work on Fevers, by Dr. Clarke, of New-castle.

"In reflecting on these appearances, are not the conclusions which force themselves upon us of the most convincing and decisive nature, as to the previous existence of excitement in the vascular system: inflammation, abscess, mortification, and gangrene; and yet not admitting them to exist as the consequences of excitement, because the sounds of *putrid* and *malignant* are ringing in our ears!!! Will the worshippers of these formidable names have the candour to tell us how they are produced? or by what means it happens that, under their most unequivocal appearances, as in the particulars referred to above, the consequences of the most active sta[illegible]s of inflammation have been found to exist."

There is much useful and practical matter in the work before us, and we cannot do less than recommend it to the perusal of our readers.

---

*Surgical Essays.* By Astley Cooper, F.R.S. Surgeon to Guy's Hospital; and Benjamin Travers, F.R.S. Surgeon to St. Thomas's Hospital.—Part I. 8vo. pp. 264. Cox and Son, 1818.

The intentions of the authors in the publication of this work are so well expressed in the Preface, and it displays so much urbanity and zeal for the improvement of surgery, that we are disposed to present it wholly to our readers.

"The publication of a work, upon the plan of that which is now submitted to the public, has been for many years the intention of the authors, and was only postponed until the junior should have succeeded to the situation of surgeon to St. Thomas's or Guy's Hospital.

"It would be impossible for any person to witness the abundant opportunities afforded by these munificent establishments, (which together accommodate more than eight hundred patients, besides numerous out-patients,) without participating in the anxious wish of the authors, that they should be adequately improved.

"The variety which of necessity occurs in the practice of the surgeons—the facility afforded to them in their respective plans of treatment—the opportunities of improving the practice of medical surgery—of observing the results, general and comparative, of operations of every description—and especially of prosecuting enquiries into morbid anatomy, by prompt examination of the dead body, and of parts removed by operation, are advantages which, while they afford ample compensation for the labours of clinical research, would allow no pretext for indifference in those, who, conscious of their value, were not influenced by an ardent desire to improve and impart them.

"It is from such impressions that the present undertaking has originated. Numerous as is the class of students attending the hospitals of the metropolis, and zealously as they are in general disposed to acquire knowledge in their profession, it falls to the lot of few to reside long at these schools of practice; and it is therefore especially desirable, that their attention during that period should be directed to the objects which are to them of greatest importance, and their judgments assisted in forming conclusions from the facts which fall under their daily observation. It is surely not less desirable, when they are settled at a distance from the seats of education, and only the sphere of private practice, limited even in its greatest extent, remains open to them, that the suspension of professional intercourse should not chill and subdue the natural ardor of their minds; that they should be enabled to keep pace with the never-ceasing progress of observation, and not forfeit their interest in the pursuit of the noblest of all practical sciences.

"It is from no presumptuous feeling that the authors of this work entertain a hope, that the publication of practical Essays, containing the best opinions they are able to form, may, in time, produce a beneficial influence upon the practice of their art, by exposing popular errors, and deciding some of the many points still involved in doubt and obscurity.

"Neither the transactions of societies, nor the periodical journals, can be expected to afford space for the details of hospital practice: seldom the narration of occurrences in one branch of the profession is interesting to more than one class of readers, and especially such a narration as includes more of the common than the rare; for it is neither in the contemplation nor desire of the editors to promulgate marvellous cases. The singularity of a case may be a good reason for its publication, but its importance is a better; and, in general, the greater its singularity, the less its importance. They are, therefore, disposed to think that occurrences, which by reason of their frequency attract less notice than they deserve, may yet be found to contain important matter for consideration.

"How much additional value the relation of an ordinary case acquires when supported and illustrated by others nearly resembling it, is too obvious to require exemplification. It is only in the assemblage and multiplication of facts that their proper bearing can be seen, and their real value ascertained; or that they can be admitted to form a safe groundwork for the superstructure of theory.

"In the present undertaking, it is perhaps needless to say that no attempt will be made improperly to bias the public judgment. The cases of failure will be as fully detailed as those of which the issue is fortunate; the errors will be as faithfully exposed as the merits of practice, and the progress of truth shall not be retarded by the disingenuous concealment of error. In pursuit of this main object, it is the determination of the writers to avoid all invitation to controversial discussion; and, while they endeavour, "studiis et rebus honestis," to deserve the good-will of their professional brethren, they will rather submit to be the subjects than the authors of critical animadversion.

"For the literary composition of the work, its nature and their avocations will, they trust, render apology unnecessary,—the value of the matter will be their more particular concern. The delineation of morbid parts will be given in the form of slight engraving, in the hope of rendering the work less expensive and more extensively useful.

"The motive which has induced the authors to execute this work conjointly, is the opportunity which it furnished of giving to the public the more extended experience derived from the practice of the two hospitals; and they hope soon to be enabled to publish a second Part, but the period of publication must for the present be left undetermined."

The work commences with an essay on *Dislocation*, by Mr. Cooper. This we shall pass over, as not being adapted for analysis: we must, however, observe, that the subject is treated with much perspicuity, and in so comprehensive a manner as to furnish the student with all the useful practical information respecting it that can be acquired in the present state of our pathological knowledge. Novelty of ideas or opinions it does not contain; but this was hardly to be expected,—we may almost say, hardly to be desired,—on the subject of dislocations.

The essay next in order is on *Iritis*, by Mr. Travers.

It is somewhat extraordinary that Iritis, a disease productive of such serious effects, and which is by no means of rare occurrence, should, until a very late period, have been so indistinctly noticed by writers on the pathology of the eye. Previously to the essay of Dr. John Adam Schmidt, published at Vienna in the year 1801,* no precise description of this disease had appeared; and the observations of this author are principally relative to it when it occurs subsequently to the operation for cataract. The remarks of Dr. Sanders on Iritis are useful only in the diagnosis and treatment; the pathology of the disease was passed over unnoticed by him.†

---

* Ueber Nachstaar und Iritis nach Staar Operationen, von Dr. Johann Adam Schmidt. Wien. 1801.

† Iritis formed the subject of the inaugural dissertation of Dr. W. F. Edwards, of Paris, in the year 1814; but the observations of this intelligent physiologist, on this occasion, are too general to

1

The essay by Mr. Travers, whose opportunities for observation have been extensive, excited a hope that our desire for more general information respecting it would be gratified: this has, however, been but imperfectly fulfilled. The remarks of Mr. Travers tend to show the obscurity in which the subject yet remains, and to prove that much attention and labour will be required for its elucidation, rather than to furnish us with any precise and satisfactory knowledge. Several points respecting it have, however, been ascertained by Mr. Travers, and his observations will contribute much to assist the researches of others; but, it appears, an attempt to deduce general conclusions from too confined and partial a series of facts, has led him into many perplexing inconsistencies. It is a subject that requires much acuteness of discrimination for its investigation, and a logical mode of reasoning in the deduction of general ideas respecting it: the ardour of our anticipation was, therefore, somewhat chilled on the perusal of the first paragraph of this essay: it does not furnish us with that accuracy of description we were disposed to expect from Mr. Travers.

"The interior tunics of the eye are subject to inflammation, arising either as an idiopathic affection, or from the extension of protracted inflammation of the superficial tunic. The effects of this inflammation are conspicuous in the iris, and by the term Iritis I mean to express the *deep-seated inflammation* of the eye."

The author then observes that—

"It appears in company with rheumatism of the chronic form, sometimes with gout; with the constitutional signs of the lues venerea; and during or following the action of mercury upon the system.

"It is in reference to the two latter associations of the iritis, that I propose principally to consider the disease in this essay.

"Although the cases of iritis co-existing with sore throat, cutaneous eruptions, and nodes, which are regarded and successfully treated as venereal, are too frequent to escape observation, the eye is not mentioned by Mr. Hunter and other pathologists as a part subject to be affected by the syphilitic poison."

We think a little careful investigation would have induced Mr. Travers to correct this assertion; for there is hardly an author, from Astruc downwards, who professed to describe all the symptoms which commonly accompany syphilis, that does not mention inflammation of the eyes as one of them. Mr. Hunter noticed this ophthalmy, but expressed doubts

---

be of much importance, and his description of the disease is by no means sufficiently precise. His thesis showed that further investigation was necessary.

respecting the nature of it: he was not disposed to attribute it strictly to the action of the venereal virus.* Scarpa has expressly described it as a symptom of syphilis. Schmidt and Edwards state, that it frequently arises from this cause.

Mr. Travers then makes the following reflections on the general circumstances under which the disease appears.

"I have met with cases in which the mercurial action had been set up for primary lues, and the iritis was present, unaccompanied by any secondary symptom of that disease. I have also met with cases of iritis in which pains confined to the joints have been accompanied by eruptions widely differing from those which have been commonly considered venereal. Its occurrence, indeed, during the use of mercury is so well established and familiar a fact, among persons who see much of ophthalmic diseases, that their first enquiry of a patient labouring under inflammation of the iris is, not whether he has recently contracted syphilis, but whether he has been taking mercury. For, although the iritis is frequently met with where no mercury has been taken, it is scarcely ever seen as a sequela of syphilis, where mercury has not been exhibited so as to affect the system; and I think it is more frequent as a consequent upon the use of mercury, than occurring as an idiopathic disease. Under this denomination I do not include those frequent affections of the choroid and iris, which are plainly depending upon the duration and extension of superficial ophthalmia."

"It appears to me at present impossible to pronounce whether the iritis, so frequently presented after sores on the genitals, and accompanied by eruptions, is the effect of a morbid poison, or of the mercurial poison; or, thirdly, the casual effect of exposure to an exciting cause in a state of predisposition from the mercurial impregnation of the system."

The few instances in which the concurrence of iritis with the constitutional symptoms of syphilis, where mercury had not been employed for the primary affection, might reasonably be expected, renders the decision of these questions extremely difficult; but, as iritis does undoubtedly appear as an idiopathic affection, there can be no reason why it may not accompany syphilis, or follow the use of mercury, without being absolutely connected with either of them. An attempt to attribute it to either of those causes, in many instances, may perplex us, but not add to our knowledge. That it appears with syphilis, where mercury has not been employed, is shown by the case related by Mr. Rose, in his Paper in the eighth volume of the Medico-Chirurgical Transactions. This is a point very difficult to be decided; for, as the author observes, "although we speak of venereal inflammation, we are not in possession of any means whereby

* Treatise on the Venereal Disease, p. 324.

to demonstrate and distinguish this from common inflammation. The lymph effused upon the iris is as probable an effect of venereal inflammation, as when it is deposited upon the periosteum."

The following remarks, with the cases illustrative of them, merit attentive consideration.

"The sympathy or consent which obtains between certain parts appears to influence the course of their diseased actions, whether simple or sui generis. The throat, skin, and bones, are observed to be affected by the poison of mercury as well as of lues, and also by that which resembles lues, except in its curableness by other means than the use of mercury. It is remarkable that the eye frequently forms a link of this chain, or, in other words, that the inflammation of the choroid and iris co-exists with affections of the throat,* skin, and bones; whether these are referred to syphilis, pseudo-syphilis, mercury, or rheumatism. It is also remarkable, that the remedy which exerts the most powerful and obvious effect upon the inflamed periosteum and skin, acts with the same remarkable efficacy upon the inflamed tunics of the eye, and *vice versâ.* Depositions beneath the periosteum, resembling nodes, and eruptions on the skin, are not less certainly induced by the poison of mercury, than is the deposition upon the choroid, ending in incurable amaurosis.

"The two following cases afford a brief explanation of the statement, that the consent between certain parts probably influences the course of diseased actions, simple or specific; and I am inclined to believe that this is the explanation of some at least of the cases resembling syphilis, which are occasionally cured without the use of mercury.

"A young gentleman of particularly correct conduct and retired habits, while upon his voyage from the West-Indies to this country, for the purpose of education, was troubled with a sore upon the penis, which occasioned him much anxiety, as he had never yet indulged in sexual intercourse, and was wholly unable to account for it. On his arrival, he applied to a confidential friend, stating the circumstance. His skin was shortly afterwards covered with an eruption, which yielded only to a course of mercury.

"A married gentleman of character, whom I well knew, applied to me with a paraphymosis and superficial ulceration behind the corona glandis; in which case I know that no venereal commerce had taken place, nor had any mercurial medicine been employed to affect the system. It began in consequence of a hair having accidentally applied itself around that part. Six weeks after the healing of the sore, the whole cutaneous surface exhibited

* "There is, in fact, a general tendency to sore throat, and even to affections of the eyes, in all the varieties of cutaneous eruptions, which are accompanied with any degree of fever."—*Bateman's Synopsis*, note, p. 333.

a plentiful crop of pustular eruptions. There were present, at the same time, an inflammation and soreness of the membrane of the fauces, severe pains in the bones and joints, and enlargement of the bursæ upon the olecrana of the elbows."

We transcribe the following paragraph to show the perplexities which result from the consideration of a disease in a confined point of view, and without having arranged the subject to be investigated in a regular manner, by which the more general facts respecting it might be first ascertained, and afterwards those which are occasional and accidental. But our reflections shall follow the paragraph.

"In considering whether mercury, acting as a poison upon the system, can be a cause of the iritis which so often follows its use, we should bear in mind that, although we rarely see the disease unpreceded by mercury, we as rarely see it unpreceded by primary symptoms of lues. It would be unfair to refer it to one rather than the other, as far as this evidence goes. But I have satisfied myself of the frequent origin of the disease while the system has been fully charged with mercury; the sore on the penis, for which it was exhibited, having long since healed, and no after-symptoms of lues being to be discovered. I have also seen it where the system had been mercurialized for gonorrhœa. During the free exhibition of calomel in strumous inflammation, I have frequently seen the iris take on the inflammatory action; and, while the cure of the iritis was daily accomplishing by the action of mercury in one eye, it has been common to observe the inflammation beginning in the other, as if the action produced effects diametrically opposite upon the sound and the inflamed part. When a person is attacked with ophthalmia, whose system is charged with mercury, the inflammation is never confined to the conjunctiva, but invariably affects the deeper tunics, so far as my observation goes. Yet I have never seen the iris affected in cases of eczema mercuriale, but always the conjunctiva of the eyelids. With what I have considered to be the mercurial sore throat (very unlike the venereal), and mercurial eruptions and nodes, I have frequently seen it in conjunction: but, with one exception, I do not call to mind a well-ascertained case of inflamed iris, during the constitutional action of mercury for a disease in which the genitals had had no concern—as enlargement of the liver, &c."

Mr. Travers, as we have informed our readers (page 505), considers that "iritis may appear in company with rheumatism of the chronic form, sometimes with gout; as an idiopathic affection; or from extension of inflammation of the conjunctiva; with the constitutional signs of the lues venerea; and during or following the action of mercury on the system." And he states, that it is in reference to the two latter associations that he proposes principally to consider the disease. It appears to us, that it is to the disposition to consider it in this restricted point of view, that we are to attribute many of the confused and unconnected notions expressed in the above paragraph. By confining his observations to the disease under peculiar circumstances, he has neglected the more general ones which determine its production, and endeavoured to explain the nature of it by observations deduced from its occasional characters. The practice of Mr. Travers having been, we presume, principally confined to London, where the existence of syphilis,—at least, where the exhibition of mercury for the supposed existence of that disease,—is so general; has led him, we imagine, to consider iritis as too intimately connected with one of the above-mentioned circumstances. Much of the obscurity thrown over the history of this disease in the above paragraph will be dispelled, on admitting, with Professor Schmidt and other living pathologists, that it may originate from the same general causes which induce common inflammation. In a word, Mr. Travers has not taken sufficient care to consider iritis, (to speak in the language of nomenclature,) as a genus of disease of which there may be several species; and thus the advice of Hippocrates, so peculiarly important in pathological investigation, to ascertain *οἷα ἐξ οἵων*, has not been strictly conformed to.

We shall pass over the description of the general appearances of the disease, as not containing any thing new of importance: there are, however, some peculiarities in it, the author thinks, when it arises from syphilis, of which we shall transcribe his account.

"The inflammation of the eye after syphilis is not, so far as I have observed, characterised by any peculiarities, so much as the shade of colour of the inflamed conjunctiva and sclerotic, and the appearance assumed by the deposited lymph. The former have a brick-dust or dusky red, instead of a bright scarlet hue; and the lymph is compact and brown, and intimately adhering to the iris, instead of curd-like, loose, and of a yellowish white colour."

Preparatory to the consideration of the mode of treatment, we meet with the following singular observations:—

"The treatment of the disease, which is fortunately one of the best-ascertained points in pathology, would seem to throw light upon its origin; but the habit of reference to the remedy, in order to ascertain and class the disease, appears to me a proceeding altogether unscientific and erroneous. To consider that the symptoms which mercury cures are *therefore* syphilitic, and that those which it does not cure are not so, is to abandon altogether the analytical investigation of disease. How many diseases, essentially different, are cured by the same remedy! How confused and useless for the purpose of diagnosis, which is the sum and substance of rational practice, would be a nosology framed upon such a basis! That the effect of remedies is important as an adjuvant to the study of nosology, may be admitted; but it can never be allowed to form a part of the *historia morbi,* much less to assume the importance of a diagnostic."

How the treatment of a disease can be considered to constitute a point of pathology, or diagnosis be the sum and substance of practice, we are utterly at a loss to imagine; and our patience has, fortunately for us, never been so severely tried, as to be obliged to confer with men who have had such "unscientific and erroneous" notions as to imagine that the effects of remedies can "throw light on the origin" of a disease. Our observations on such an occasion would be marked with more notes of admiration than the above expressions of Mr. Travers.

Several cases are related which favour the opinions of Mr. Travers respecting the most frequent causes of the disease, previously to his general reflections on the mode of treatment. This, as our readers will anticipate, principally consists in blood-letting, and the use of mercury, joined with the application of belladonna, as directed by Mr. Sanders.

Whether surgeons in general will admit of the following claims on the part of those of the London Infirmary for Diseases of the Eye; and our continental neighbours acknowledge the truth of what is expressed in the note; may be doubted. We can only observe, that we have seen them regularly employ mercury for the cure of iritis connected with syphilis, as the most obvious remedy.

"The beneficial use of mercury in iritis, strange to say, is an observation of but few years' date.* Its use was still more recently confined to the cases which were combined with traces of the syphilitic poison. But, averse as are European practitioners, from education or prejudice, or both, (for they are not always unconnected,) to introduce mercury into the system during a state of active inflammation, it is now, by a multitude of facts, incontestibly established as a remedy of unfailing efficacy in the most acute form and in every variety of inflammation of the iris. The ascertainment and promulgation of this fact are due to the Infirmary of this metropolis for Diseases of the Eye; and in the catalogue of modern contributions to medical science, except the practice of vaccination, I know of none entitled to rank before it."

Some ingenious remarks ensue on the mode of action of mercury, which our limits will not permit us to enter into

* "Its effect is but partially known on the continent: I am sorry that I have it not in my power to refer, in proof of this opinion, to a modern Latin tract, on the Inflammations of the Eye, which I some time ago perused."

the consideration of. We must terminate the present subject with the following candid observations of the author:—

"I am quite aware, that the facts which I have stated in this paper admit of different conclusions. Some may incline to consider the iritis after mercury as casual, and not essentially depending upon its use. Others may be unwilling to yield their belief of the presence of syphilis in the system, for which they will conclude that the remedy had been ineffectually administered. The subject is obscure, and an earnest desire to promote the investigation of it is perhaps the best apology I can offer for submitting it in so unfinished a state to the profession."

The ensuing paper is on a *Case of Ligature on the Aorta*, by Mr. Cooper. "I fear," observes the author, "that the title of this paper may impress the reader with an idea that nothing could justify me in performing the operation which I am about to describe; for that a ligature made upon the aorta must necessarily prove fatal. But I trust that it will be seen in the sequel, that the operation was not attended with the immediate danger which might have been apprehended; that the patient complained of but little pain during its performance; that it afforded the only hope of safety; and that we had to lament, not that the operation was performed, but that it had not been sooner done."

These conciliatory remarks may impress deeper on our minds the idea we may have of the philanthropic disposition of the author, but they are unnecessary as an address to surgeons. No one deserving of that title can doubt of the propriety of the operation in the present instance.

We shall present our readers with an abridged account of the case, and detail the circumstances which ensued from the operation.

Charles Hutson, a porter, aged 38 years, was admitted into Guy's Hospital, April 9, 1817, for a swelling in the left groin, situated partly above and partly below Poupart's ligament; which was found to be an aneurism. Thirteen months previous to his admission, he had fallen against the corner of a chest, which struck him on the groin. Some degree of swelling soon after appeared in the groin, which continued to increase until the time of his admission into the hospital. On the third day after this, the swelling increased to double its former size, and the pulsation became less distinct, excepting in the course of the iliac and femoral arteries. Pressure was applied on the fore-part of the swelling for about a fortnight, when the skin over the aneurism showed a disposition to slough; and, after a fortnight longer, slight hæmorrhage took place from the sac of the aneurism, a portion of the integuments covering it having sloughed away. The hæmorrhage recurred in a slight degree on the following day, and on the next to a profuse degree; so that the patient appeared nearly exhausted, and his fæces were passed involuntarily. Mr. Cooper attempted to tie the artery from within the sac; but this was found to be impracticable. The only chance, then, that appeared to remain of preventing his immediate dissolution from hæmorrhage, was by tying the aorta.

To be enabled to effect this, an incision was made into the linea alba, three inches in length, extending equally above and below the navel; a slight curve was given it, to avoid the umbilicus. An aperture was then made into the peritoneum, and the finger introduced into the abdomen, when the incision of that membrane was extended to nearly the same length as that of the external integuments. The finger was then passed between the intestines, and an opening scratched with the nail through the peritoneum on the side of the aorta; the finger was then passed behind the aorta, and made to penetrate the peritoneum on the opposite side of the vessel. A single ligature was passed around it, by means of a blunt aneurismal needle, and the edges of it brought out at the external wound.

"During the time of the operation, the fæces passed off involuntarily, and the patient's pulse, both immediately and for an hour after the operation, was 144 in a minute; he was ordered thirty drops of tincture of opium and camphorated mixture, and the involuntary discharge of fæces soon after ceased. I applied my hand to his right thigh immediately after the operation, and he said that I touched his foot; so that the sensibility of that leg was very imperfect.

"For the following particulars I am indebted to Mr. Cox, one of my apprentices.

"At midnight his pulse was 132.

"26.—At one o'clock in the morning, the patient complained of heat in the abdomen, but he felt no pain upon pressure; he said, that his head felt hot, and that he had pain in the shoulders. His lower extremities, which were cold soon after the operation, were now regaining their heat; his body was in other parts covered with a cold sweat. The sensibility of the lower extremities has been very indistinct since the operation.

"At two o'clock, he felt so comfortable from his medicine that he wished to have more of it, and ten drops of tincture of opium were given him: his legs were wrapped in flannel, bottles of hot water were applied to his feet, and he then said that the heat of his belly was lessened.

"At six o'clock, the sensibility of his limbs was still imperfect.

"At eight o'clock A.M. he expressed himself as feeling quite comfortable; he, however, passed no urine, and had no evacuation; his right limb was warmer than the left, and the sensibility was returning.

"At noon, the temperature of the right limb was 94; that of the left, or aneurismal limb, 87½.

"At one o'clock P.M. Mr. Cooper visited him; and, as he walked up the ward, he appeared much gratified at seeing his patient, who was at the point of death the evening before, and who was now adjusting his bed-clothes, and smiled as Mr. C. approached his bed.

"At three o'clock, after a fit of coughing, the man was much alarmed with the idea of the thread having slipped into the wound: it was a false alarm; but, to prevent the idea of its occurrence, it was fastened to a quill. Soon after this he complained of pain in the abdomen; it was not very severe, nor did it last long, readily yielding to fomentations. As he had no evacuations, he was ordered an enema.

"At six o'clock P.M. he vomited, soon after the glyster had been administered: the heat of the right leg was 96; that of the left, or diseased limb, 87½.

"At nine in the evening he took half a glass of port wine in warm water, which he immediately rejected: he complained of pain in the loins; his pulse was 104, and feeble; he was very restless, and had an involuntary discharge of fæces.

"Eleven o'clock at night, his pulse 100 and weak; he still vomited.

"27.—At 7 A.M. the report was, that he had passed a restless night; the vomiting had returned at intervals: his pulse 104, weak and fluttering; he complained of pain all over his body, more particularly in his head; and the carotids beat with considerable force. He had great anxiety expressed in the countenance, was very restless; and the urine dribbled from him, with some degree of pain at the end of the penis.

"At eight o'clock A.M. the aneurismal limb appeared livid and felt cold, more particularly around the aneurism, but the right leg remained warm.

"At eleven o'clock, his pulse was 120 and weak; he appeared to be sinking. To the questions which were put to him he did not return any answer; he appeared to have an uneasiness about the heart, as he kept his hand upon the left breast.

"He died at eighteen minutes after one, P.M. having survived the operation forty hours.

"After being informed of his death, I requested Mr. Brookes of Blenheim-street to attend with me at the inspection of the body. Mr. Travers, surgeon of St. Thomas's Hospital, Mr. Stocker, apothecary of Guy's, and a large concourse of medical students, attended the examination.

"When the abdomen was opened, we found not the least appearance of peritoneal inflammation, excepting at the edges of the wound. The omentum and intestines were free from any unnatural colour; the edges of the wound were glued together by adhesive inflammation, excepting at the part at which the ligature projected. We were much gratified to find that the ligature had not included any portion either of the omentum or intestine: the thread had been passed around the aorta about three-fourths of an inch above its bifurcation, and about an inch or rather more below the part at which the duodenum crossed the artery. Upon carefully cutting open the aorta, a clot of more than an inch in extent was found to have sealed the vessel above the ligature; below the bifurcation, another, an inch in extent, occupied the right iliac artery, and the left was sealed by a third, which extended as far as the aneurism: all were gratified to observe the artery so completely shut in forty hours. The aneurismal sac, which was of a most enormous size, reached from the common iliac artery to below Poupart's ligament, and extended to the outer side of the thigh. The artery was deficient from the upper to the lower part of the sac, which was occupied by an immense quantity of coagulum.

"Upon consideration of all the circumstances of the case, to what are we to attribute this man's death? It did not arise from inflammation, for the viscera of the abdomen were perfectly free from it.

"His death appears to me to have been owing to want of circulation in the aneurismal limb; for, although the warmth of the other limb was preserved, that on which the aneurism was seated never gained its natural heat, which must have arisen from the great bulk of the aneurism, and from the disturbed state of the coagulum which it contained, which would prevent the free course of the blood through the aneurismal sac. That limb never recovered its natural heat, there being seven degrees difference between the two extremities; the sensibility also in the right limb was returning, which did not appear to be the case in the left. In an aneurism, therefore, similarly situated, the ligature must be applied before the swelling has acquired any very considerable magnitude."

Such are the reflections of Mr. Cooper on this subject; and from them it would appear that some important circumstances respecting it were overlooked by him. The cause he has assigned for the death of the patient is by no means satisfactory: should it not have been sought for in the brain? The pain in the head, forcible beating of the carotids, and vomiting at intervals, seem to indicate that the immediate cause of death would have been found in the derangement that had taken place in the functions of that organ, consequent on the forcible propulsion and increased afflux of blood to it, produced by the stoppage of the circulation in so large a proportion of the body. The patient, it is true, lost a considerable quantity of blood previous to the operation; but it does not appear probable that the quantity was sufficient to counterbalance the circumstance to which we have alluded; and the whole of the symptoms that ensued seem to show the correctness of this opinion.

We may almost remark, that we see no reason why the ligature might not have been applied on the common trunk of the iliac arteries.

Mr. Cooper quotes the accounts of the cases inserted in the Medico-Chirurgical Transactions, and in Mr. John Bell's Surgical Observations, in which obliteration of the aorta in the human body was discovered after death; instances of which are also related by Scarpa: but they cannot prove any thing with respect to the propriety of the application of a ligature to the aorta. In those cases, the obliteration, it is most probable, came on gradually; consequently, the small anastamosing arteries dilated so as freely to carry on the circulation in the lower extremities before that in the large trunks had ceased: a very different circumstance from the sudden stoppage of it by means of a ligature on the trunk of those vessels.

The effects of ligatures on the aorta of dogs are not so strictly applicable to the human body as Mr. Cooper seems to infer. The great difference in the proportion of the size of the extremities to that of the rest of the body in that animal, when compared with those of man; and the comparatively trifling consequences which ensue from disturbance of the functions of the brain in all the inferior classes of animals; are sufficient to prevent any satisfactory conclusions to be drawn from those experiments. M. Beclard, who has frequently made this experiment, observes, that the aorta can be tied in dogs without making an opening into the cavity of the peritoneum: and he also states that, although some of them recovered from it, yet the greater proportion died of hæmorrhage, on the separation of the ligature.

The next essay is on *Phymosis and Paraphymosis*, by Mr. Travers. The following passages will shew the object of the author: we need only observe, that those who have neglected to make themselves acquainted with the proper mode of practice in these diseases from books, or who have not been taught it by experience, will find much useful information in the essay of Mr. Travers.

"It has upon many occasions appeared to me, that practitioners are too anxious to contend with the specific character of the venereal disease, to the neglect of the inflammatory state of the affected parts, and of the evils which mercury produces when exhibited during its height.

"The abuse of administering mercury for an acute gonorrhœa and recent sores, accompanied by phymosis, or an approach to that state, is of common occurrence, and it is far from being recognised by the profession as an established rule of practice, that its constitutional administration is inadmissible during the existence of active inflammation in cellular textures. The gangrenous inflammation, which spreads and destroys with such terrible rapidity the entire integuments of the lower opening of the pelvis in young and delicate females, proceeds, I am convinced, from the exhibition of mercury for inflamed and irritable sores, oftener than from any other cause.

"There is no class of cases exhibiting the local effects of impure intercourse in so formidable a view, in its nature so wholly distinct from a syphilitic taint, in its origin so purely casual, as that which forms the subject of this paper. That it was not formerly so regarded, nor even by writers comparatively modern, it would be easy, if it were necessary, to show. It is true that the mischievous consequences which I shall presently detail rarely occur in private practice; a plain proof that they are the result of neglect or gross ill-treatment, and that such complaints in a recent state are easily remediable. But, in hospital and pauper practice, we continually meet with people under the constitutional influence of mercury, who are subjects of inflammation of the penis, bordering on gangrene; often by their own indiscreet conduct; and sometimes, it must be confessed, by following the advice of persons who have the credit of being better informed."

An essay on *Exostosis*, by Mr. Cooper, constitutes the most valuable part of this volume. The extensive experience of the author has furnished him with ample series of facts, which he has arranged with much perspicuity; and, although this essay does not contain any thing new of much importance respecting the mode of treatment, yet it will assist us in the diagnosis, and add to our knowledge of the pathology, of the disease.

"Exostosis (the author observes) has two different seats; it is either *periosteal* or *medullary*. By the *periosteal* exostosis, I mean a deposition seated between the external surface of the bone, and the internal surface of the periosteum, adhering with firmness to both surfaces; and by the *medullary* is to be understood a formation of a similar kind, originating in the medullary membrane and cancellated structure of the bone.

"With regard to its nature, exostosis is of two kinds, either *cartilaginous* or *fungous*. By the *cartilaginous* is intended to be expressed that species which is preceded by the formation of a cartilage, which forms the *nidus* for the ossific deposit; and by the *fungous* is to be understood a tumor of softer structure than cartilage, yet firmer than fungus in other parts of the body, containing spicula of bone, malignant in its nature, depending on a peculiar state of constitution and action of vessels,—a disease similar to that which Mr. Hey has denominated *fungus hæmatodes*, but somewhat modified by the structure of the part in which it originates.

"The venereal exostosis, or node, although depending upon a different cause, is still a cartilaginous exostosis. But this subject I do not now intend to consider, as it ought rather to form part of an essay on the venereal disease.

"I know of no bone in the body which is not liable to the formation of these diseases, although there are some in which it much more frequently occurs than in others.

"Upon the bones of the cranium we see both kinds of exostosis. That which forms between the outer table of the skull and the pericranium, is of an extremely hard consistence, is generally attended with little pain, and does not usually acquire any considerable magnitude; but a very large tumor, with a basis of bone, was lately removed by Sir Everard Home from the head of a person in St. George's Hospital. Four of these have been known to arise from the same os frontis; one of larger, and three of smaller size. The fungous exostosis springing from the diploë of the skull is of less firm consistence, and is endowed with a greater degree of vascularity, than the former. It is of a malignant kind, and is found to proceed through the inner table of the skull, occasioning disease of the dura mater; and, by its pressure upon the brain, to produce a diseased state of the functions of that organ, by which means life is destroyed."

The *fungous exostosis of the medullary membrane*, the author continues to observe, begins with a general enlargement of the limb in the part opposite to the seat of the complaint, and to a considerable extent around it. When it has attained considerable magnitude, although it produces some diminution of the power of motion of the limb, it seldom occasions pain. When pain does arise, it is of an obtuse kind, and extends in the course of the bone and nerve; and it becomes very acute when a nerve happens to be stretched by it, which sometimes takes place in exostosis of the thigh-bone, which presses on the sciatic nerve.

The general health is, in these cases, defective; paleness, debility, and an irregular state of the bowels, mark the early stages. The limb, at length, becomes of considerable size at the affected part, but the skin retains its natural colour. The tumor feels hard in many parts; in others, it is elastic, and conveys, from the touch, the idea of a fluid; but, on its being punctured, nothing issues excepting blood. Its surface next becomes tuberculated, and some degree of tenderness is felt. To these appearances succeed constitutional irritation; the rest becomes broken, the appetite impaired, and the bowels irregular.

During the continuance of these symptoms, many weeks elapse; and, at length, ulceration takes place on the tubercles. When the tumor itself becomes exposed, it discharges a bloody serum; a fungus then arises, which bleeds occasionally, and, as is usually observed in fungous diseases, the blood is loose in its coagulation, and a large quantity of serum is separated from it. The bleeding relieves the pain for a short time. Sloughing at length takes place, by which considerable portions of the swelling are separated; but it is never completely destroyed by gangrene. The loss of blood, and the constitutional irritation, at length wear out the patient; but the period is often prolonged to several years.

It often happens, in this disease, that tumors of a similar kind form in other parts of the body, during its progress.

On cutting through this species of exostosis, it appears composed of lobulated masses, of various colours and consistence. Some parts are like yellow fat, others resemble brain, and others are composed of coagulated blood. In some parts, the white substance is found nearly as firm as cartilage; but it is generally of a more spongy appearance, and contains spicula of bone. The shell of bone itself is, in some parts, absorbed; in others, it is only thinner than usual: in some, it has been seen immensely expanded, so as to form a case like wire-work over the tumor.

Of the cause of this disease, Mr. Cooper observes, nothing certain is known. It has, in some instances, been attributed to a blow; in others, to a jump from a considerable height: in the lower jaw, it has appeared to arise from a diseased tooth.

It appears requisite that an unhealthy state of the system should exist, for the above-mentioned causes to produce the diseased action which follows the injury.

The disease is frequently remedied in its early stage, by correcting the general diseased state of the system; and for this purpose, the oxymuriate of mercury, with the compound decoction of sarsaparilla, has been found most efficacious The local treatment consists in the application of leeches, and blisters kept open with savine ointment. Tying the artery which supplies the tumor with blood has not been found productive of benefit, when the disease has made considerable progress; excision, or amputation, is the only resource.

In the *cartilaginous exostosis of the medullary membrane*, the shell of the bone becomes expanded, or, rather, the original shell is absorbed, and a new one deposited; and within the ossific cavity thus produced, a large mass of firm, elastic, fibrous cartilage is found. It appears to arise from common inflammation in a constitution even not unhealthy, when irritation has been kept up for a length of time. It has sometimes appeared to arise from a decayed tooth.

In the treatment of this disease, the source of irritation is to be sought for; the removal of which, although it may have advanced to a considerable extent, has sometimes prevented its further progress. Should this, however, prove insufficient, it will be proper to remove the external shell of bone by a saw, and dislodge the cartilage it contains with an elevator. If the integuments be carefully preserved, but little deformity follows this operation.

*The fungous exostosis of the periosteum* hardly differs in its symptoms from the fungous exostosis of the medullary membrane, except that the general swelling of the limb is less, and the particular tumor more prominent. There is the same want of sensibility in the early stages of the disease, with some pain afterwards; the skin remains for a long time free from discolouration, but at length assumes the same tuberculated appearance as in the species to which we have alluded.

This tumor is generally found, on dissection, covered with thickened periosteum, within which a white elastic substance is discovered, having numerous small spicula of bone passing in radii from the surface of the original bone, the shell of the bone in a great degree remaining: this, however, is sometimes removed by absorption. A slight degree of inflammation appears to have existed within the cancellated structure in some instances; and small portions of ossific matter are usually found deposited in its cells.

This disease is usually attributed to external injuries; but any irritation of a bone, in an unhealthy state of the system, will produce it. In the collection at Guy's Hospital, there is a specimen of it, where it arose from external exfoliation of the os femoris: between the periosteum and the bone, in this case, instead of the cartilaginous process which accompanies internal exfoliation, an immense fungus is thrown out between the periosteum and the surface of the bone; showing that the nature of the inflammation is determined by the existing state of the constitution, and that a very unusual effect may be produced by a frequent cause of irritation.

The mode of treatment of this disease is similar to that required in the fungous exostosis of the medullary membrane; but it is only in the first dawn of it that benefit can be derived from medicine.

*Cartilaginous exostosis between the periosteum and bone* originates in inflammation of the periosteum and corresponding part of the bone; from which a deposition of cartilage, of a firm texture, takes place, adhering to both those surfaces. Osseous particles are then formed in this cartilage, which are at first thrown out from the original bone, but are afterwards secreted in the cartilage: for it appears that cartilage is constantly secreted between the periosteum and bony mass, which constitutes the exterior surface of this tumor. When these tumors cease to enlarge, and the disease has been of long standing, they are found to consist, on their exterior surface, of a shell of osseous matter, similar to that of the original bone of the same cancellated structure, and communicating with the original cancelli of the bone. When the tumor is deprived of its phosphate of lime, the cartilaginous structure retains its original form and magnitude; the ossific matter appears to be deposited in the same manner as in healthy bone.

This disease is accompanied with but little pain at its commencement; but, when it has acquired considerable magnitude, it becomes productive of inconvenience, and is sometimes attended with ulceration of the skin. The most frequent seat of it is upon the inner side of the femor, just above the condyle, and in the direction of the insertion of the triceps muscle. It occurs also on the tibia, immediately under the insertion of the sartorius and gracilis muscles; it is often seen on the os humeri, at the insertion of the deltoid muscle.

The cause of that variety of the disease which has but a small base, the author conceives, is exertion disproportionate to the strength of the subject. The tendons which are fixed in the bone becoming sprained, inflammation takes place in them, which is thence communicated to the periosteum and bone; a deposition of coagulable lymph is consequently produced in the direction of the sprained and inflamed tendons.

The periosteal exostosis may admit of relief from internal medicine, from external applications, and, when considerably advanced, from a surgical operation. It is only in the first stage that internal medicine is serviceable; the most efficacious is mercury in small doses, with decoction of sarsaparilla. Stimulating plasters, as the ammoniacal plaster with mercury, may be applied to the part. When the tumor has attained a considerable size, and is productive of great inconvenience, it may be removed by the saw, and without much pain or danger. The disease is not often found to return; and, if the integuments are carefully dissected on either side, the wound generally heals in a favourable manner. It appears that bones, after operations, unite by adhesion to the soft parts; if adhesions in any instance cannot be produced healthy granulations rise from the surface of the bone, and cicatrization takes place upon these, as upon other parts of the body.

This volume concludes with an essay *on Wounds and Ligatures of Veins*, by Mr. Travers; in which the author relates the results of some researches he has made on this interesting and hitherto obscure subject.

After having noticed the cases on record of death ensuing from inflammation of veins, Mr. Travers proceeds to adduce some facts which favour the opinions he has been led to form, from the results of his own observations and enquiries. He first shows that inflammation of a vein, which was stated by Mr. Hunter to be always present in those vessels when they passed through parts in a state of violent inflammation, appears to arise, in some instances, merely from sympathy with other parts; an opinion which is supported by preparations in the collection of Mr. Langstaff, of Basinghall-street, in which "the veins in the vicinity of parts destroyed by the phagedena of the malignant fungus, and diseases of this class, are filled by a soft pulpy matter, resembling in texture the destructive growth."

The cases related in the third volume of the Medico-Chirurgical Transactions, by Mr. Wilson, and by Dr. Clarke in his Essays, are next referred to, as showing the disposition veins have to be affected with continuous inflammation. Inflammation of the veins after bleeding is very common in horses, which is attributed by Mr. Coleman to the indisposition of the integuments of that animal to adhesive inflammation; and thence the cavity of the vein is very liable to be exposed to the influence of the air, and the orifice of the wound in the vessel affected with ulceration.

After making a comparison between the anatomical character of arteries and veins, the author proceeds to trace their pathological distinction.

"1. The coats of arteries (he observes) are subject to ossific inflammation and deposition to an unlimited extent. Those of veins are very rarely the seat of this, and never, so far as I have observed, continuously, as we see in arteries.

"2. In their disposition to inflame, as well as in other particulars, veins resemble absorbents; but the cases of inflamed vein bear no comparison, in point of frequency, to those of inflamed absorbents. It has been observed, that a wound of the same extent, and inflicted with the same instrument as that made in blood-letting, does not cause the extensive inflammation which we occasionally see it produce in veins, in any other texture of the body. This, however, if we consider the frequency of venesection and the rarity of these cases, can scarcely be admitted. The cellular membrane, the fascia, and especially the absorbents, inflame at least as frequently as the veins, and not less extensively from slight injuries, for example, from venesection, and from small wounds of the extremities.

"3. The most material fact, however it may be explained, is this:—the inner or lining membrane of veins is subject to diffused or continuous inflammation; that of arteries very rarely, if ever. I am aware that a preternatural redness of the interior surface is sometimes observed to run through the course of an artery.

"4. The inner membranes of arteries and veins are susceptible of the adhesive inflammation. That of the former is defined, whether excited by pressure or by wound, or occurring spontaneously. I never saw the internal coat of an artery furred with lymph; and even where lymph is deposited in quantity sufficient to obstruct the current of blood, the deposit occupies a narrowly-defined space; and the inflammation, by whatever cause excited, or however acute, is similarly circumscribed. In veins, on the contrary, the inflammation extends from the point of irritation towards the heart, or from branch to trunk. The lymph coats the vein like a fringe; and, though the quantity effused is sometimes sufficient to obstruct the tube, the inflammation is often not bounded by the obstruction.

"5. The inner coat of veins is susceptible of the suppurative inflammation, and the inflammation is often mixed, presenting both terminations alternately, viz.—in lymph and pus. That of arteries is, I believe, incapable of suppuration, unless in a state of ulceration.

"6. Veins are more disposed to ulcerative inflammation than arteries. I know not whether the interior tunics of either are capable of granulating, nor am I able to say whether there exists a difference in their power of resistance to gangrene.

"7. The contrasted character of the inflammation of arteries and veins above mentioned, explains the active constitutional sympathy peculiar to the latter. This corresponds with our observation of the difference in this respect presented by the bounded and the undefined inflammation of joints, the peritoneal or the pleural cavities, and the other shut sacs of the body.

"8. The constitutional symptoms excited by inflamed veins resemble in type those of diffused inflammations in other organs. They are similar to those of inflamed absorbents, which vessels also resemble the veins in their disposition to continuous inflammation."

The opinions of Bichât respecting the relative disposition of veins to inflammation with that of arteries, are next examined; and facts are adduced by Mr. Travers in opposition to the opinions of that eminent physiologist, who considered that veins were more disposed to that action than arteries. Mr. Travers next details the results of his observations on the mode in which re-union takes place in wounds of the veins.

"When a vein is wounded longitudinally or obliquely, there is no separation of the edges of the incision; so that only a little, if any, blood trickles from the aperture, unless pressure be made nearer to the heart, to obstruct the passage of the blood in the vessel. If, therefore, an animal be killed immediately after a wound of the vein, from which no blood has been drawn, the lips of the wound will be found in contact; and, if permitted to live for a short time, the cicatrix will form a line.

"If a vein is opened by a transverse section, it bleeds without the addition of pressure; and, if the vein is half divided, the hæmorrhage is with much difficulty suppressed,—the blood escaping into the cellular sheath of the vein and of the contiguous muscles in the direction of the current, and forming a distinct coagulum between the orifice and the external wound. The longitudinal or oblique wound, by which blood has issued in quantity, presents the same appearances: an oval naked coagulum forms the plug of the orifice, and a flattened covered clot, which is an extravasation into the cellular sheath, extends to some distance around it. At the end of twenty-four hours, the lips of the wound are found separated, forming an oval proportioned to the length of the incision, the edges everted and adhering to those of the clot; the eversion seeming to be the effect of distension from the extravasation into the sheath: there is no blush upon the edges, nor any appearance of organisable or secreted lymph in the vein or the wound. At three days, the same appearances are observed; but the internal margin of the wound is somewhat elevated and rounded, and a thin and narrow membraneous expansion is perceived to be continuous with the everted edge of the internal tunic. The clot itself is more compact; and, upon section, presents concentric lamellæ, the interior being of a lighter colour than the exterior layers.

"On the fifth day, these appearances are more confirmed: the membraneous appearance extends over the surface of the clot, if the wound is not exceeding a quarter of an inch in length; and in larger wounds the coagulum, which is reduced in size, has a membraneous surface. On the eighth day, the new membrane is complete, the interior margin of the wound is raised and tumid, and the coagulum of a common bleeding wound is nearly absorbed. From the twelfth to the sixteenth day, numerous vasa vasorum may be seen, by the aid of a glass, passing from the internal tunic over the newly-formed membrane, and anastomosing upon it. At the latter period the edges are less raised, so as to be more upon a level with the new membrane, and have a slight red blush. The coagulum is entirely absorbed.

"On the twentieth day, it is only possible to distinguish the recent from former wounds, by the tenuity, smoothness, and transparency, of the new membrane, compared with the old, which is dense, tough, and wrinkled."

A ligature on a vein, he observes, does not divide the internal tunic, but draws it into longitudinal folds. The portion next the heart is perfectly empty and collapsed; that next the extremity is distended by a long, and generally firm, coagulum of blood. There is no blush upon the inner tunic; much less any sign of adhesive inflammation, or thickening of the proper coats of the vein, or agglutination of the contiguous folds: but the cellular sheath is thickened by a deposit of coagulable lymph, in the vicinity of the ligature. At the end of seven days, the effusion of lymph into the cellular sheath is increased so as to encompass the ligature. After nine days, the ulceration of the coats has begun. The completion of this process is generally effected at the end of from fifteen to twenty-five days.

After some further observations of less importance on this subject, and a comparison of these appearances with those produced on arteries by the same causes, Mr. Travers enters into the consideration of the causes of inflammation of the veins.

"The indisposition of the venous membrane to inflame is not, as appears to me, inconsistent with its obvious tendency, under adequate excitement, to inordinate and excessive inflammation. It is not unusual to find the morbid action of parts that are difficultly roused least controlable, when once set up. The mixed terminations of the inflammation of the venous membrane in the adhesive, suppurative, and ulcerative states, and its disposition to spread by continuity, are characteristic of inflammation in the cellular membrane, as seen in the erysipelas and other affections; and are, therefore, probably to be referred to the predominance of this texture in its composition.

"It appears difficult to account for the origin of this inflammation: if we refer it to the operation of any peculiar and purely local cause, how are we to reconcile the infrequency of its occurrence after an operation so constantly and so carelessly practised as that of blood-letting?—not to speak of other operations and accidents, by which these parts are wounded, lacerated, contused, compressed, ulcerated, &c. Besides, we have seen that it occasionally follows various and dissimilar modes of local irritation. The apparent inadequacy of the local injury as a cause, the rapid and violent character of the inflammation, and the high constitutional disorder which is manifested, would, on the other hand, rather induce us to ascribe it to a peculiar state of the constitution; and that subsequent venesections, performed upon these patients, have been unattended by similar local effects, cannot be admitted as an objection. Yet, allowing the rarity of the case and the frequency of the operation, it will be found, in nine instances out of ten, to ensue upon a local injury, however simple. Exposure of the cavity of the vein is a circumstance often not accompanying the injuries by which the inflammation is induced, and continually occurring, without any ill consequence. Non-adhesion and festering of the wound in the integument is an effect of suppuration beneath it, as frequently, I should think, as a cause. The subcutaneous abscess, as is observed by Mr. Hunter, is of no moment, *if the vein and parts below have united;* in the human subject, abscess of the wound, and diffused inflammation of the subcutaneous cellular texture, the lymphatics and their glands, and even the fascia, producing œdematous swelling and tension of the entire limb, are certainly more frequent than the inflammation of the vein as a consequence of venesection, where the wound has been improperly treated or neglected, and the patient suffered to use his arm without restriction. It appears, from the experiments related, that the wound of a vein after blood-letting does not take on direct adhesion, but that the last effused blood forms a plug or stopper in the wound; that the agglutination is complete between the clot and the edges of the orifice which it occupies, and that the clot serves as a bed for the production of the new membrane. If from any cause suppuration occurs, the clot will be loosened and displaced, and the action which commonly ensues where the adhesive is defeated or disturbed, viz.—the ulcerative, will be set up on the margin of the orifice.

"The ulcerative inflammation upon the edges of the wound destroys the process by which the reparation is effected, and which, by the eversion of the edges and the presence of the clot, is rendered in a degree extraneous to the canal; and it is therefore a cause of irritation abundantly sufficient for the excitement of continuous inflammation, to which we see that this membrane is so remarkably disposed. Thus 'the imperfection of union is continued on to the cavity of the vein;' and, if the presence of a thrombus in excess, the breaking of the agglutination, and the necessary displacement of the clot by secondary bleedings, casual or designed, the immediate and unguarded exercise of the limb, the friction of the wound in the skin, or the application of adhesive plasters, which fret and inflame it, are either or any of them adequate causes of the suppurative inflammation in the vicinity of the wound,—they are, I imagine, beginners of the mischief, though it may often be prevented from reaching the interior of the vein. I think, however, that the mode in which the wound is stopped and repaired renders more intelligible the effect of these disturbances, and the manner in which, from its exposed condition, the vein is liable to be involved in the inflammation: in short, that, if the wound healed like that of an artery, it would not be subject to a secondary inflammation."

When veins become obliterated, the author observes, it is not, as in the case of arteries, from a union of their internal surfaces: the vein becomes filled with a coagulum, an interstitial deposit takes place between its coats, by which the canal becomes gradually closed, and the vein is rendered a round cord of cartilaginous hardness.

Inflammation of veins appears to have two modes of progress and termination: the formation of pus, and sometimes of abscesses in the veins, which, by ulceration of its sides, communicate with the cellular membrane, and point externally in the course of the vessel; and continuous, pure adhesive inflammation, without any production of matter. In the latter, the inflammation produces a continuous deposition of lymph, which extends to the large trunks, and sometimes reaches the heart; the system becomes affected with hectic fever, which ends in exhaustion. In the former, the fever is typhoid, speedily producing delirium, and terminating fatally in a few days. The author concludes with the following reflections:

"There have been many conjectures respecting the cause of the fatal termination of these cases, at which I confess I feel surprised. Among others, the inflammation by extension of the heart, or the membranes of the brain, and the conveyance of pus into the circulation, have been mentioned. If we consider the importance of the veins in the economy, the extent of surface which the collective areæ of the venous trunks afford, (larger, I imagine, than any of the shut sacs of the body,) and the diffused and disorganizing character of the inflammation, we can surely be at no loss to account for the disturbance of the system. It is an error to suppose that any quicker sympathy exists between the constitution and the venous, than the arterial or absorbent system. I say this, because I have observed something like that superstitious alarm, which is excited by events that we do not expect and cannot explain, has been produced by the fatal catalogue of tied veins, and a comparison of this with the generally successful cases of tied arteries. All the mystery of veins is, as I have attempted to shew, that they are indisposed to inflame; but, when excited, inflame by continuity, and therefore it is that the constitution sympathises so deeply. The same would happen if arteries were subject to a similar law; and it is fortunate for mankind that this is not the case. Tying or dividing the vein is not a cure for varix; but, if it were, the cure of a disease, which is little more than an inconvenience, would be too dearly bought at a risk of life. It is not so with aneurism.

"It is my intention to prosecute this investigation; and I shall feel sincerely thankful for any communications connected with a subject of so much intricacy and importance."

On taking a revisal of our comments upon this work, we find they have extended beyond what we anticipated or is strictly appropriate to the limits of our Journal; yet we are not disposed to extenuate any part of them which contain the observations of the authors,—a sentiment which discloses, more perfectly than any express terms we could use, our opinion that it contains much useful information: but we must remark, this is not information of the high character we were inclined to expect from Mr. Cooper and Mr. Travers; and the promises made in the preface are yet to be accomplished. In this we, however, imagine some degree of propriety may be discerned: this volume is the commencement of a series, and the authors may have considered that it were better " Non fumum ex fulgore, sed ex fumo dare lucem."

---

### FOREIGN MEDICAL AND PHYSICAL SCIENCE AND LITERATURE.

### *Transactions of the Physico-Medical Society of New York.* Vol. I.

